# Decreased translation of *Dio3* mRNA is associated with drug-induced hepatotoxicity

**DOI:** 10.1042/BJ20130049

**Published:** 2013-06-13

**Authors:** Kate M. Dudek, Laura Suter, Veerle M. Darras, Emma L. Marczylo, Timothy W. Gant

**Affiliations:** *Systems Toxicology Group, Medical Research Council Toxicology Unit, Hodgkin Building, Lancaster Road, Leicester LE1 9HN, U.K.; †Institut for Chemistry and Bioanalytics, School of Life Sciences, University of Applied Sciences and Arts Northwestern Switzerland (FHNW), Gründenstrasse 40, 4132 Muttenz, Switzerland; ‡Laboratory of Comparative Endocrinology, Department of Biology, Section Animal Physiology and Neurobiology, KU Leuven, Naamsestraat 61, PB 2464, Leuven, B-3000, Belgium; §Centre for Radiation, Chemical and Environmental Hazards, Public Health England, Harwell Campus, Didcot, Oxfordshire OX11 0RQ, U.K.

**Keywords:** InnoMed PredTox consortium, iodothyronine deiodinase type III, liver, polysome profiling, toxicogenomics, translation, ALT, alanine transaminase, AST, aspartate aminotransferase, Cy3, indocarbocyanine, Cy5, indodicarbocyanine, D1–D3, iodothyronine deiodinase type I–III (encoded by *Dio1*–*Dio3* respectively), GAPDH, glyceraldehyde-3-phosphate dehydrogenase, lcRNA, long non-coding RNA, qRT-PCR, quantitative reverse transcription–PCR, RXR, retinoid X receptor, T_2_, 3,3′-diiodothyronine, T_3_, 3,5,3′-triiodothyronine, rT_3_, reverse T_3_, T_4_, thyroxine, TBST, Tris-buffered saline with 0.05% Tween 20, TH, thyroid hormone, TR, TH receptor

## Abstract

Recent work has demonstrated the importance of post-transcriptional gene regulation in toxic responses. In the present study, we used two rat models to investigate mRNA translation in the liver following xenobiotic-induced toxicity. By combining polysome profiling with genomic methodologies, we were able to assess global changes in hepatic mRNA translation. *Dio3* (iodothyronine deiodinase type III) was identified as a gene that exhibited specific translational repression and had a functional role in a number of relevant canonical pathways. Western blot analysis indicated that this repression led to reduced D3 (the protein expressed by *Dio3*) levels, enhanced over time and with increased dose. Using Northern blotting techniques and qRT-PCR (quantitative reverse transcription–PCR), we confirmed further that there was no reduction in *Dio3* mRNA, suggesting that translational repression of *Dio3* is an important determinant of the reduced D3 protein expression following liver damage. Finally, we show that drug-induced hepatotoxicity appears to cause localized disruptions in thyroid hormone levels in the liver and plasma. We suggest that this leads to reduced translation of *Dio3* mRNA, which results in decreased D3 production. It may therefore be possible that this is an important mechanism by which the liver can, upon early signs of damage, act rapidly to maintain its own energy equilibrium, thereby avoiding global disruption of the hypothalamic–pituitary–thyroid axis.

## INTRODUCTION

The control of gene expression downstream of transcription is of physiological and toxicological importance due to the speed at which this level of regulation can be used to generate new proteins [1]. Such responses are essential within the CNS (central nervous system), for example, where cells demonstrate rapid variation in their metabolic activity [2,3], and within the liver where a rapid response is necessary to combat the constantly changing chemical milieu. Translational regulation enables a faster, more flexible response to cellular stress because the need to transport newly synthesized mRNAs to make more protein is avoided [3]. This response is demonstrated in cases of temperature shock and DNA damage where the majority of mRNA translation is shut down, with just a few key mRNAs increasing their rates of translation to enable the cell to deal with the additional stress [4]. Analysis of the extent to which mRNAs are recruited to ribosomes gives a quantifiable measure of translation efficiency and is a well-established technique [5]. This process can be coupled with genomics methodologies to allow a global assessment of the translational activity of mRNAs following cellular stress. Furthermore, in combining these data with those obtained from transcriptional studies, a comprehensive overview of the gene-regulatory processes used by the cell to adapt to, and recover from, stress can be obtained. This approach has demonstrated a role for both transcriptional and translational regulation in many pathophysiological states such as drug resistance and cell-cycle control [6,7]. In addition, with miRNAs (microRNAs) and other non-coding RNA species already established as translational regulators [8], research focusing on those regulatory processes occurring at the level of mRNA translation has increased. In fact, in certain cases, this level of control is the primary determinant of gene expression [2,9,10].

To investigate whether such translational mRNA regulation was important following drug-induced hepatotoxicity, we used two *in vivo* models. One of these was developed by the European Union Innovative Medicines Initiative (InnoMed) PredTox consortium. The consortium undertook a series of experiments to investigate liver injury in the rat following dosing with novel pharmaceutical reagents that had failed during development due to overt toxicity [11]. The other was the well-established rat thioacetamide model [12,13], which we have included to supplement and verify the findings from the PredTox model. Using polysome fractionation and microarray methods, we were able to globally analyse differential mRNA translation, and through subsequent pathway analysis with Ingenuity IPA software (Ingenuity® Systems), we explored canonical pathways of potential interest and relevance in cases of hepatotoxicity. We identified *Dio3* (iodothyronine deiodinase type III) as one of the genes that exhibited specific translational control under conditions of hepatic toxicity.

*Dio3* encodes an enzyme (D3) that is vital for TH (thyroid hormone) regulation; the maintenance of TH levels throughout life is of fundamental importance. The two major THs are T_4_ (thyroxine), secreted by the thyroid gland, and its biologically active form, T_3_ (3,5,3′-triiodothyronine). In combination, these two molecules play a critical role during development, cellular proliferation and metabolic homoeostasis [14–16], and even transient disruptions in their levels can cause a wide number of transcriptional gene changes [17]. Prolonged deficiency causes growth retardation, impaired cognitive function and, in severe cases, infantile haemangioma [18,19]. In addition to D3, there are two further deiodinases (D1 and D2), all of which are highly conserved throughout the vertebrate kingdom [20]. Of the two THs, T_4_ is the most abundant; it is transported through the cellular membrane, via membrane transporters, before diffusion to the nucleus where it is converted into the more active T_3_ by D1 and D2 [21]. On a tissue-specific basis, if levels of T_3_ and T_4_ become too high, then D3 is recruited from the plasma membrane [22], and, together with D1, it converts T_3_ and T_4_ into the inactive metabolites rT_3_ (reverse T_3_) and T_2_ (3,3′-diiodothyronine) respectively. Levels of D3 are T_3_-dependent, requiring the presence of TR (TH receptor) α [23]; this feedback mechanism is vital for the control of both the hypothalamic–pituitary–thyroid axis [24,25] and TH levels throughout the body.

*Dio3* shows particularly high expression in fetal and placental tissues [26,27], where it functions to protect the fetus from high levels of maternal TH. The majority of adult tissues demonstrate only low expression of *Dio3*; however, studies show that the rat brain maintains high D3 activity throughout life [28]. These data supports a functionally protective role for the enzyme, in this instance in modulating thyroid levels within neurons. In addition, in some circumstances, there is re-expression of *Dio3* in the adult, for example during proliferation and cell growth [29]. This has been demonstrated in a number of pathophysiological conditions including cancer, myocardial infarction and liver regeneration following partial hepatectomy [19,30,31].

Although much is known about the transcriptional regulation of *Dio3*, its translational, or post-transcriptional, regulation has not been extensively investigated. Levels of *Dio3* are increased transcriptionally in the presence of THs [32], retinoic acid [33] and growth factors [34], and reduced by growth hormones [35] and in hypothyroidism. Genomic imprinting of *Dio3*, via differentially methylated regions [36], is also suggested to be a contributor to transcriptional regulation [32]. Although not yet identified in the case of *Dio3*, there are published data on the other deiodinases that suggest that post-transcriptional regulation is essential; for example, *Dio2* demonstrates significant post-transcriptional regulation [37]. Within the brain, the translational regulation of *Dio2* is critical for the preservation of TH levels to prevent hypothyroidism, and it has been shown that the increases in D2 activity are far greater than can be accounted for by transcriptional changes in *Dio2* mRNA [38]. Within the rat and human adult liver, *Dio2* is not expressed [39]; however, *Dio3* is. We therefore investigated whether within the liver this important mode of regulation could be controlled by *Dio3* rather than *Dio2*.

In the present study, we show that *Dio3*, like *Dio2*, can show a change in expression at the post-transcriptional level. We hypothesize that this mechanism may play a key role in the synthesis of D3 protein, the control of TH levels and the maintenance of the hypothalamic–pituitary–thyroid axis in cases of drug-induced cellular stress to the liver.

## EXPERIMENTAL

### Animals: PredTox

With a view to reducing the financial and time restraints caused by the extensive testing required for any new drug candidate, a consortium comprising 15 pharmaceutical companies, two SMEs (small and medium enterprises) and three universities was set up in 2005. This consortium, the InnoMed PredTox project, formed under European Union Framework Programme 6, sought to develop genomic biomarkers for the early detection of drug-induced toxicity. Male Wistar rats received pharmacologically active doses of 14 compounds that had failed in development owing to hepato- and/or nephro-toxicity. The treatment regime involved dosing for up to 15 days with a vehicle dose, a low dose or a high dose of each compound. The five agents that caused the most pronounced hepatobiliary injury were selected for further investigation in the present study. Liver samples from rats treated with these five compounds were provided as donations from the PredTox consortium. The compounds and their properties are shown in Supplementary Table S1 at http://www.biochemj.org/bj/453/bj4530071add.htm.

### Animals: thioacetamide model

Male Wistar rats (3 months, 300–350 g) were dosed via the i.p. (intraperitoneal) route with Thioacetamide (Sigma) dissolved in 0.9% saline to final doses of 50 mg/kg, 100 mg/kg and 150 mg/kg. These doses fall below the threshold for overt toxicity [12,13]. Vehicle-only-treated rats were used as controls. Six animals were used at each dose level and all Figures show means±S.E.M. for each measurement. After 24 h, half of the animals from each group were anaesthetized using isoflurane and blood was withdrawn via the descending vena cava. Blood was collected into lithium/heparin tubes and the plasma was isolated. Organs were perfused *in situ* with PBS and animals were killed by decapitation under terminal anaesthesia. Organs were harvested, sections were taken for histological analysis and the remainder were snap-frozen in liquid nitrogen. The remaining animals from each group underwent the same process after a further 24 h. All procedures were licensed under U.K. Home Office project licence 80/2126.

### Measurement of ALT (alanine transaminase) and AST (aspartate aminotransferase) levels

To measure plasma levels of ALT and AST, kits from Sentinel Diagnostics (Alpha Laboratories) were used according to the manufacturer's instructions.

### Translational profiling

Sucrose density fractionation was used to separate mRNAs according to their ribosomal mass. mRNAs that are under active translation will have a large number of ribosomes attached [40], increasing their density and resulting in them migrating towards the bottom of the gradient; those that are less actively translated, with fewer ribosomes attached, will be less dense and will therefore be situated towards the top of the gradient. Approximately 150 mg of each liver sample was ground to a powder in liquid nitrogen using a pestle and mortar and then lysed in 15 mM Tris/HCl (pH 8.0), 300 mM NaCl and 15 mM MgCl_2_, plus inhibitors [1 mg/ml heparin, 100 μg/ml cycloheximide and 80 units of RNasin® (Promega)]. Following centrifugation at 12000 ***g*** for 5 min at 4°C, the resulting supernatant was added to a 10–50% sucrose gradient and ultracentrifuged at 388000 rev./min in a Beckman ultracentrifuge for 2 h using a SW40 rotor. Sucrose of a greater density was used in combination with a Harvard syringe pump system to divide each gradient into ten to 16 individual 1 ml fractions. These fractions were collected into tubes containing 1 ml of Tri Reagent (Sigma), and RNA was extracted according to the manufacturer's instructions.

### Genomic analysis of mRNA fractions

**Figure 1:**
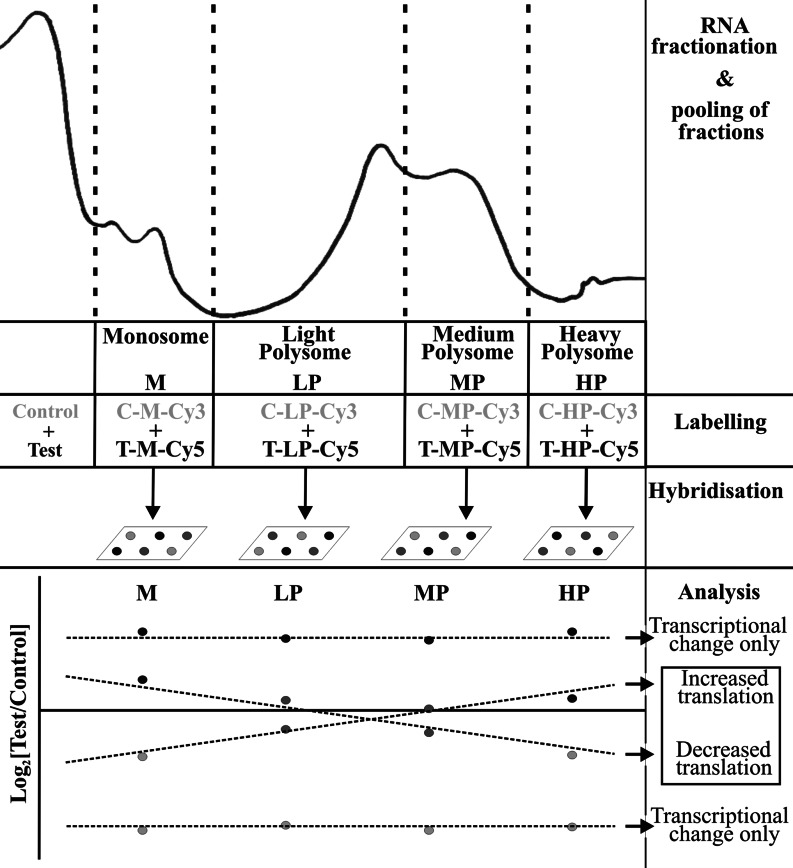
Translational profiling of liver samples taken from rats treated with the PredTox compound FP014SC RNA from the livers of control (vehicle-treated) and test (compound-treated) animals was fractionated by sucrose density centrifugation and pooled into four fractions: monosome (M), light polysome (LP), medium polysome (MP), and heavy polysome (HP). Each of the four control (C) fractions were labelled with Cy3 and hybridized against each of the four corresponding test (T) fractions labelled with Cy5. The statistical significance of differences between C and T in each of the four fractions was calculated using a reverse-labelled two-tailed Student's *t* test [41]. All data with four *P* values >0.05 were rejected. Linear regression analysis was then performed on the four log_2_ ratios of test fractions/control fractions. This showed movement of mRNAs through the gradient and enabled differentially translated mRNAs to be identified. Reverse-labelling reactions were also performed and incorporated into the analysis.

The individual fractions were subpooled into four groups, monosomal, light polysomal, medium polysomal and heavy polysomal (Figure 1), according to ribosomal density. Microarray analysis was performed by hybridizing control (vehicle-treated) monosomes against test (high-dose-treated) monosomes on one microarray, control light polysomes against test light polysomes on a second microarray, and so forth for each subpool of fractions (Figure 1). Equal amounts of RNA from each subpool were precipitated with 10 μl of 3 M sodium acetate (pH 5.2) and 275 μl of 100% ethanol at −20°C overnight. Following centrifugation at 12000 ***g*** for 10 min at 4°C, pellets were washed twice with 75% ethanol and resuspended in 10 μl of water. The precipitated RNA was reverse-transcribed, hydrolysed and coupled to a dUTP-conjugated Cy3 (indocarbocyanine) or Cy5 (indodicarbocyanine) dye (GE Healthcare). The labelled cDNA samples were mixed and hybridized at 42°C overnight to a 70-mer MEEBO (Mouse Exonic Evidence-Based Oligonucleotide) microarray (http://www.microarray.org/sfgf/meebo.do). The microarrays were printed in-house using an ArrayJet Ultra Marathon microarrayer and an Illumina probe set (Invitrogen), which covers the entire mouse genome. We had established previously that these microarrays showed excellent cross-species reactivity between mouse and rat (T. Gant, unpublished work). Following overnight incubation, the slides were washed [wash 1: 1× SSC (1× SSC is 0.15 M NaCl/0.015 M sodium citrate) and 0.03% SDS for 5 min; wash 2: 0.2× SSC for 3 min; and wash 3: 0.05× SSC for 3 min] and scanned on a 4200A Axon scanner (Molecular Devices). Experiments were performed in duplicate, incorporating a dye-swap technique for *n*=5 pairs of samples. Once all hybridization reactions were complete, the results were normalized by LOWESS (locally weighted scatterplot smoothing), which uses locally weighted regression to smooth scattered data (S. Zhang, personal communication) and tested for statistical significance using a two-tailed Student's *t* test as described previously [41]. There were a maximum of four values for each mRNA, corresponding to the proportional representation of it within each subpool of fractions. By calculating the change in values, i.e. degree of slope, across the monosomal, light polysomal, medium polysomal and dense polysomal region, it was possible to determine any translational shift. In addition, the overall change in transcriptional activity across the gradient was calculated for each mRNA. The full dataset has been submitted to GEO (Gene Expression Omnibus) under accession number GSE38807. Pathway analysis of differentially translated mRNAs was performed using Ingenuity IPA software.

### Western blotting

Tissue samples were lysed at 4°C in lysis buffer [50 mM Tris/HCl (pH 7.4), 150 mM NaCl, 5 mM EDTA, 1% Nonidet P40, 0.25% sodium deoxycholate and protease inhibitors]. Protein (30 μg) was separated on a 10% acrylamide gel and transferred on to a nitrocellulose membrane (GE Healthcare). Membranes were blocked with 10% (w/v) Marvel non-fat dried skimmed milk powder in TBST (Tris-buffered saline with 0.05% Tween 20) and incubated at 4°C overnight with the primary antibody anti-Dio3 (1:1000 dilution) (Novus Biologicals) in 5% Marvel. This antibody has previously been validated [42,43] against the only other available anti-D3 antibody [44]. Following overnight incubation and washing in TBST, membranes were incubated with the appropriate horseradish peroxidase-conjugated anti-rabbit secondary antibody (1:1000 dilution), (Santa Cruz Biotechnology) and visualized using ECL (enhanced chemiluminescence) Western blotting detection reagents (GE Healthcare). To check for equal loading, membranes were stripped using 62.5 mM Tris/HCl (pH 6.8), 2% (w/v) SDS and 100 mM 2-mercaptoethanol, re-blocked and incubated overnight with anti-GAPDH (glyceraldehyde-3-phosphate dehydrogenase) (1:30000 dilution) (Sigma). Western blots were quantified using the gel analysis function of ImageJ software (NIH). To analyse time-course and dose–response effects, one-way ANOVA with Tukey's multiple comparison post-hoc test was performed using GraphPad Prism (version 5.01). Results are expressed as means±S.E.M. *P*<0.05 was considered significant.

### RNA isolation and qRT-PCR (quantitative reverse transcription–PCR)

RNA was extracted from <100 mg of liver samples using Tri Reagent according to the manufacturer's guidelines. RNA quality was assessed using an Agilent Bioanalyser, where a RIN (RNA integrity number) score of >8.5 was used as an indication that RNA was of a suitable quality for further work. To prevent genomic DNA contamination, 10 μg of RNA was treated with Turbo DNase (Applied Biosystems) according to the manufacturer's instructions. The DNase was inactivated through re-extraction of the RNA, as described above. RNA (1 μg) was reverse-transcribed using a high-capacity reverse transcription kit (Applied Biosystems) and then qRT-PCR was performed using an ABI Prism® 7000 Sequence Detection System. Primers against *Dio3* and *Actb* (β-actin) (housekeeping gene) were designed using Primer Express version 2.0.0 (Applied Biosystems) and ordered from Sigma–Aldrich. Primer sequences are given in Supplementary Table S2 at http://www.biochemj.org/bj/453/bj4530071add.htm. All primers were tested for percentage efficiency before use [45]. Reactions were performed in 25 μl of *Power* SYBR® Green PCR Master Mix (Applied Biosystems), using 450 nM each of forward and reverse primer.

### Northern blot analysis

An equal volume of RNA from each fraction was loaded on to a denaturing agarose gel as described previously [46] and, following overnight electrophoresis, was transferred on to MagnaGraph nylon transfer membrane (Labtech International) by capillary transfer (10× SSC). The membrane was cross-linked with 120 mJ/cm^2^ UV for 60 s. In addition, fractionated RNA was mixed with denaturing buffer [59% (v/v) deionized formamide, 24% (v/v) formaldehyde and 0.14 M Mops], dot-blotted on to the same membrane and cross-linked as described above. Probes derived from PCR products to the coding regions of *Dio3* and *Actb* (primer sequences given in Supplementary Table S2) were labelled with ^32^P using *Escherichia coli* Klenow fragment, and incorporation was measured by scintillation counting. Probe (10^6^ d.p.m./ml) was added to the pre-hybridized [50% (v/v) deionized formamide, 6× SSC, 5× Denhardt's solution (0.1% Ficoll 400/0.1% polyvinylpyrrolidone/0.1% BSA) and 1% (w/v) SDS] membranes and hybridized for 48 h at 42°C. The hybridized membranes were visualized using autoradiography and quantified using ImageQuant TL (version 2003.03). The value for each gradient fraction was expressed as a percentage of the total for the whole gradient.

### Determination of T_3_ and T_4_ concentrations

The levels of T_3_ and T_4_ in the liver and plasma were determined by highly sensitive and specific RIAs, as described in detail in [47,48].

## RESULTS

### Assessment of liver damage

**Figure 2:**
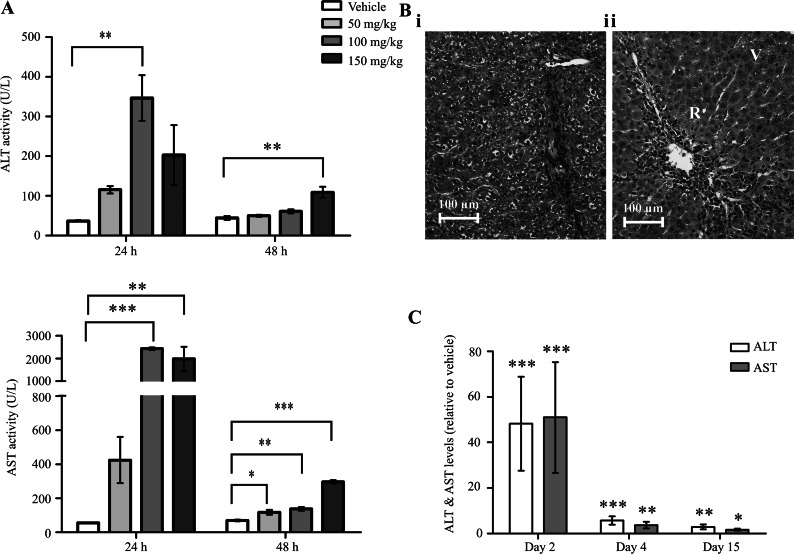
Clinical chemistry and histopathology indicate hepatotoxicity following treatment with thioacetamide and the PredTox compounds (**A**) Plasma ALT and AST levels (in units/litre) and (**B**) histopathological images 48 h after treatment with (i) vehicle-only and (ii) 150 mg/kg thioacetamide. V, vacuolation, indicative of hepatocellular injury; R, area rich in hepatocytes forming parenchymal nodules, indicative of enhanced regenerative activity. (**C**) ALT and AST levels relative to control animals from the plasma of rats treated with the PredTox compound FP014SC. Results are means±S.E.M. (*n*=3). *P≤0.05, **P≤0.01, ***P≤0.001 (ANOVA with Dunnett's post-hoc test to compare groups).

Previous work by the PredTox consortium indicated that the five compounds selected for further analysis in the present study caused hepatic injury, as determined by histopathology and clinical chemistry (Table 1). We supplemented the PredTox model with the thioacetamide-treated rat model. To determine the degree of liver injury in this additional model, plasma ALT and AST levels were measured 24 h after administration of 100 mg/kg thioacetamide. Enzyme levels were increased more than 10 (ALT) and 43 (AST) -fold when compared with the vehicle-treated samples (Figure 2A). Levels of both enzymes in the treated animals were lower at 48 h than at 24 h, reflecting the acute nature of the hepatic injury and subsequent initiation of repair mechanisms.

**Table 1:** Summary of the histopathological data for each of the PredTox compounds Severity levels: +++>++>+.

Framework Programme 6 study name	Compound sponsor	Toxicological response within liver	Severity
FP004BA	Bayer	Bile duct damage	**++**
		Hepatocyte necrosis	
		Regenerative hyperplasia	
		Inflammatory responses	
		Cholestasis	
		Fibrosis	
FP005ME	Merck	Hepatocellular apoptosis	**+**
		Necrosis	
		Peribiliary inflammation	
		Fibrosis	
		Bile duct proliferation and necrosis	
		Hepatocellular hypertrophy	
FP007SE	Boehringer Ingelheim	Increased transamininases, ALP and bilirubin	**++**
		Pericholangitis with bile duct hyperplasia	
		Cholestasis	
		Inflammation	
		Hepatocellular hypertrophy	
		Vacuolation	
FP013NO	Novartis	Absence of hepatocellular glycogen deposits	**+**
		Increased fatty deposits	
FP014SC	Bayer Schering Pharma (formerly Schering)	Severe acute necrotic liver injury	**+++**
		Elevated liver transaminases	
		Cholestasis	
		Hepatocellular vacuolation and hypertrophy	
		Regeneration and increased mitosis	

Clinical chemistry data were supported by histopathological analysis. Representative images from the livers of rats treated with the vehicle and the highest dose (150 mg/kg) of thioacetamide are shown in Figure 2(B). The images from the treated livers demonstrated some fibrosis and inflammation; the hepatocytes had small dense nuclei, indicative of hepatic injury, and there was also evidence of hepatocyte vacuolation (Figure 2B, indicated by V). However, there were also signs of some regeneration (Figure 2B, indicated by R) after 48 h, concurrent with the decreased levels of serum transaminases.

ALT and AST levels were similarly increased following treatment with the PredTox compounds. Representative data from the most severe hepatotoxic agent (FP014SC) is given in Figure 2(C). As with the thioacetamide, this effect was transient and levels had returned to close to those seen in the vehicle-treated samples by 15 days after treatment.

The histopathological summary for each of the PredTox compounds indicated a histology similar to that seen in the livers of the thioacetamide-treated rats, with common features including apoptosis, inflammation, cholestasis and regenerative repair (Table 1).

### Microarray analysis indicated that D3 is involved in drug-induced hepatotoxicity

**Figure 3:**
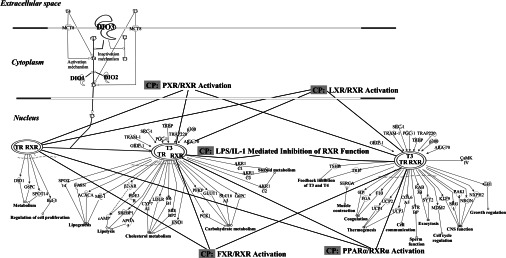
Networks of the TR/RXR activation pathway All mRNAs that demonstrated a change in translational efficiency were subject to pathway analysis using Ingenuity® IPA software. TR/RXR was identified as a key pathway. Associated RXR canonical pathways are also highlighted (‘CP’): FXR, farnesoid X receptor; IL-1, interleukin 1; LPS, lipopolysaccharide; LXR, liver X receptor; PPARα, peroxisome-proliferator-activated receptor α; PXR, pregnane X receptor. *Dio3* plays a regulatory role in the TR/RXR pathway via the control it exerts over thyroid levels.

Translational microarray analysis was performed on RNA from those PredTox samples treated with the most potent hepatotoxic agent, FP014SC (Table 1), at the highest dose and latest time point (1120 mg/kg, day 15). Analysis of polysome-associated mRNAs showed an alteration in the numbers of ribosomes recruited to individual mRNAs, indicative of changes in translational efficiency. All mRNAs that demonstrated a shift in ribosomal occupancy across the gradient (Supplementary Table S3 at http://www.biochemj.org/bj/453/bj4530071add.htm) were subjected to pathway analysis using IPA software. From this analysis, 169 canonical pathways were identified as having two or more altered genes (Supplementary Table S4 at http://www.biochemj.org/bj/453/bj4530071add.htm). Pathways under the control of RXR (retinoid X receptor) were highlighted because RXR plays a fundamental role in many disrupted metabolic pathways. Individual examination of RXR-based canonical pathways identified *Dio3* as an indirect regulator of RXR, via the control it exerts over TH levels. This is demonstrated in the TR/RXR activation pathway (Figure 3); other related canonical pathways (indicated by CP) are also highlighted. *Dio3* displayed a shift from the heavy polysomal region, to the lighter monosomal region following treatment with FP014SC (Supplementary Table S3), reflecting decreased ribosomal occupancy. This indicated that *Dio3* was less actively translated following treatment with FP014SC.

### D3 is down-regulated following drug-induced hepatotoxicity

**Figure 4:**
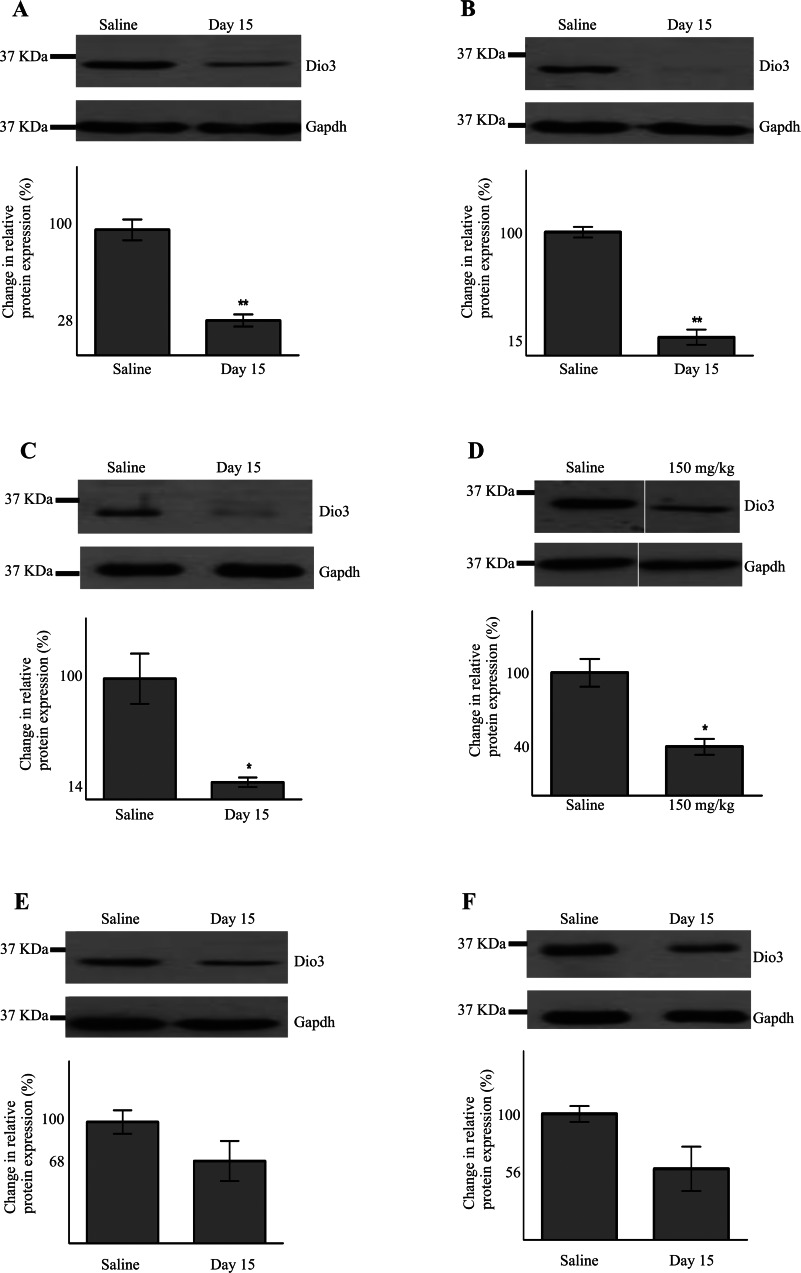
D3 protein is down-regulated following drug-induced liver damage Western blot analysis was performed on lysates from the livers of rats treated at high-dose levels with various hepatotoxic agents. D3 protein levels were normalized to those of GAPDH. Representative gel images are shown for PredTox compounds FP004BA (**A**), FP007SE (**B**), FP014SC (**C**) and thioacetamide (**D**). Non-significant reductions were seen with PredTox compounds FP005ME (**E**) and FP013NO (**F**). Mean relative levels of D3 are indicated beneath each gel image. The position of a 37 kDa protein is indicated. Results are means±S.E.M. (*n*≥3). *P≤0.05, **P≤0.001 (Student's *t* test).

**Figure 5:**
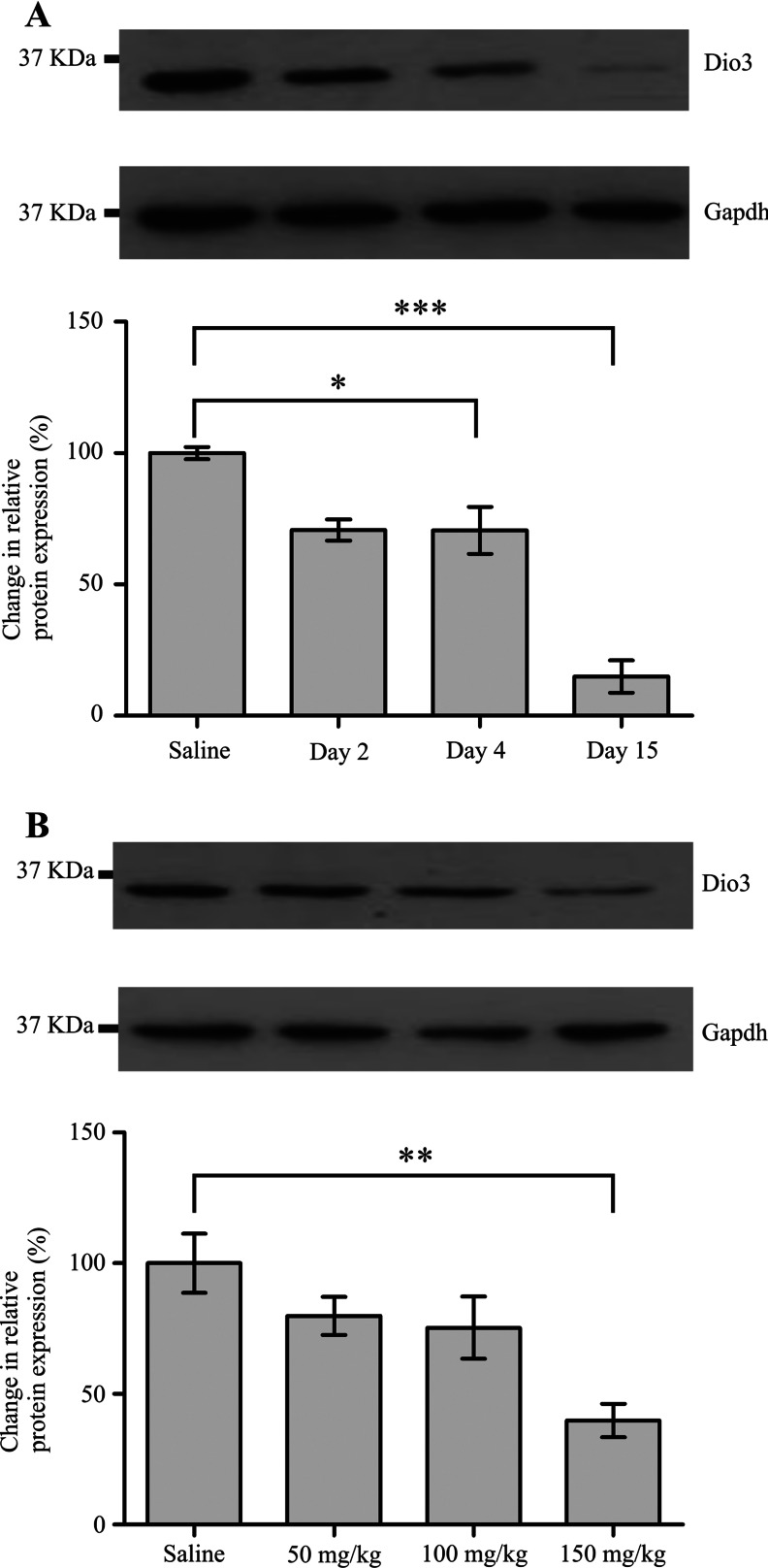
D3 protein is down-regulated in both a time- and dose-dependent manner Western blot analysis was performed on lysates from the livers of rats treated with various hepatotoxic agents. D3 protein levels were normalized to those of GAPDH. Representative gel images are shown for (**A**) PredTox compound FP007SE and (**B**) thioacetamide. Mean relative levels of D3 are indicated beneath each gel image. The position of a 37 kDa protein is indicated. Results are means±S.E.M. (*n*=3). *P≤0.05, **P≤0.01, ***P≤0.001 (ANOVA with Dunnett's post-hoc test to compare groups).

Reduced translational efficiency of an mRNA should cause a corresponding decrease in protein levels. We carried out Western blot analysis to determine D3 levels in the livers of rats treated with the PredTox compounds or thioacetamide. Following high-dose treatment for 15 days with FP004BA, FP007SE and FP014SC (Figures 4A–4C), there was a significant reduction in D3. In addition, high-dose treatment (150 mg/kg) with thioacetamide for 48 h (Figure 4D) caused significantly decreased D3 levels. A similar trend was seen with the other PredTox compounds, FP005ME and FP013NO (Figures 4E and 4F), although they failed to reach statistical significance. When considering samples on an individual basis, there was a correlation between the extent of liver damage, according to clinical and histopathological grading, and reduction in D3 levels. We therefore also tested whether dose- and time-course-related responses were evident. To measure time-related effects, we used the PredTox sample that had shown the largest reduction in D3 levels (FP007SE) following high-dose treatment for 15 days. The reduction in D3 was exacerbated over time, until, by day 15, levels were reduced to less than 15% of the vehicle-treated samples (Figure 5A). We used the thioacetamide model to look for a dose–response effect in the reduction of D3 with hepatotoxicity. Although the only statistically significant reduction in D3 was seen following treatment at the highest dose (150 mg/kg), there was a small, but consistent, change at the lower doses (Figure 5B). Two of the other PredTox compounds caused a similar effect (Supplementary Figure S1 at http://www.biochemj.org/bj/453/bj4530071add.htm).

### The reduction in protein was not due to changes in the level of *Dio3* mRNA

**Figure 6:**
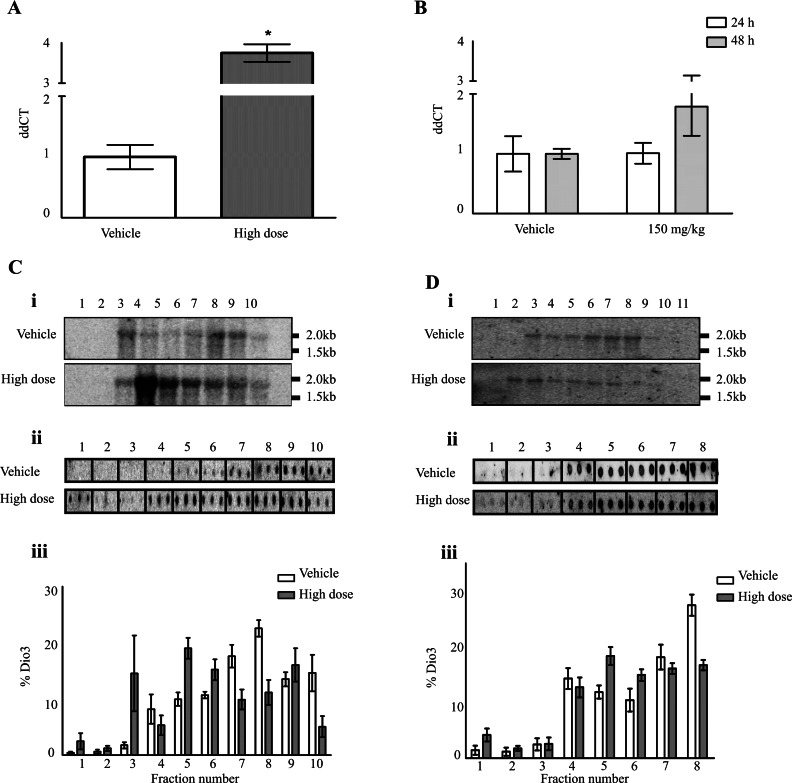
The reduction in D3 protein is due to changes in mRNA translation not transcription qRT-PCR analysis was performed on RNA extracted from the livers of rats treated with high doses of (**A**) PredTox compound FP007SE for 15 days or (**B**) thioacetamide. Results are means±S.E.M. (*n*=3). *P≤0.001 (Student's *t* test). Polysomal association of *Dio3* was measured by Northern blot analysis. Liver samples from rats treated with high doses of either (**C**) FP007SE (15 days) or (**D**) thioacetamide (48 h) were lysed and fractionated using a 10–50% sucrose gradient. RNA was extracted from individual fractions and electrophoresed on a denaturing agarose gel, before being transferred on to a nylon membrane (i). Sizes are indicated in kb. RNA from each fraction was also dot-blotted on to a nylon membrane (ii). All membranes were probed with a ^32^P labelled probe specific to *Dio3*. The proportional representation of *Dio3* within each fraction was calculated. Representative images are shown for both compounds and results are the mean±S.E.M. (*n*=3) amount of *Dio3* within each fraction as a percentage of the total (iii).

To confirm that changes in D3 at the protein level were occurring independently of a change in transcriptional activity, we performed qRT-PCR on total RNA. This showed either no change in *Dio3* mRNA levels or, in the case of FP007SE, a statistically significant increase in *Dio3* mRNA levels (Figure 6A). High-dose thioacetamide treatment caused a non-significant increase 48 h after dosing (Figure 6B) and the other PredTox compounds resulted in no change in *Dio3* mRNA levels (Supplementary Figure S2 at http://www.biochemj.org/bj/453/bj4530071add.htm).

### Less D3 was recruited to the polysomes following drug-induced hepatotoxicity

Using the FP007SE and thioacetamide studies, we performed Northern blot analysis to verify that changes were occurring post-transcriptionally, at the translational level. Hybridizing with a radiolabelled probe enabled the proportional representation of *Dio3* across the density gradient to be determined. We saw an increase in *Dio3* mRNA abundance in the lighter fractions of the compound-treated samples compared with the vehicle-treated samples (Figures 6C, i, and 6D, i), indicating lower translational efficiency. A similar validation technique was performed by machine dot-blotting RNA from each fraction across the gradient on to a nylon membrane and hybridization with the same *Dio3*-specific probe. As with the initial Northern blot analysis, when quantified, the treated samples demonstrated a shift from the heavier density fractions to the lighter density fractions (Figure 6C, ii, and 6D, ii). This supported the hypothesis that the changes in protein levels were due to fewer ribosomes being recruited and therefore less efficient translation of *Dio3* mRNA.

### The liver and plasma had reduced levels of T_3_ and T_4_ following toxic injury

**Figure 7:**
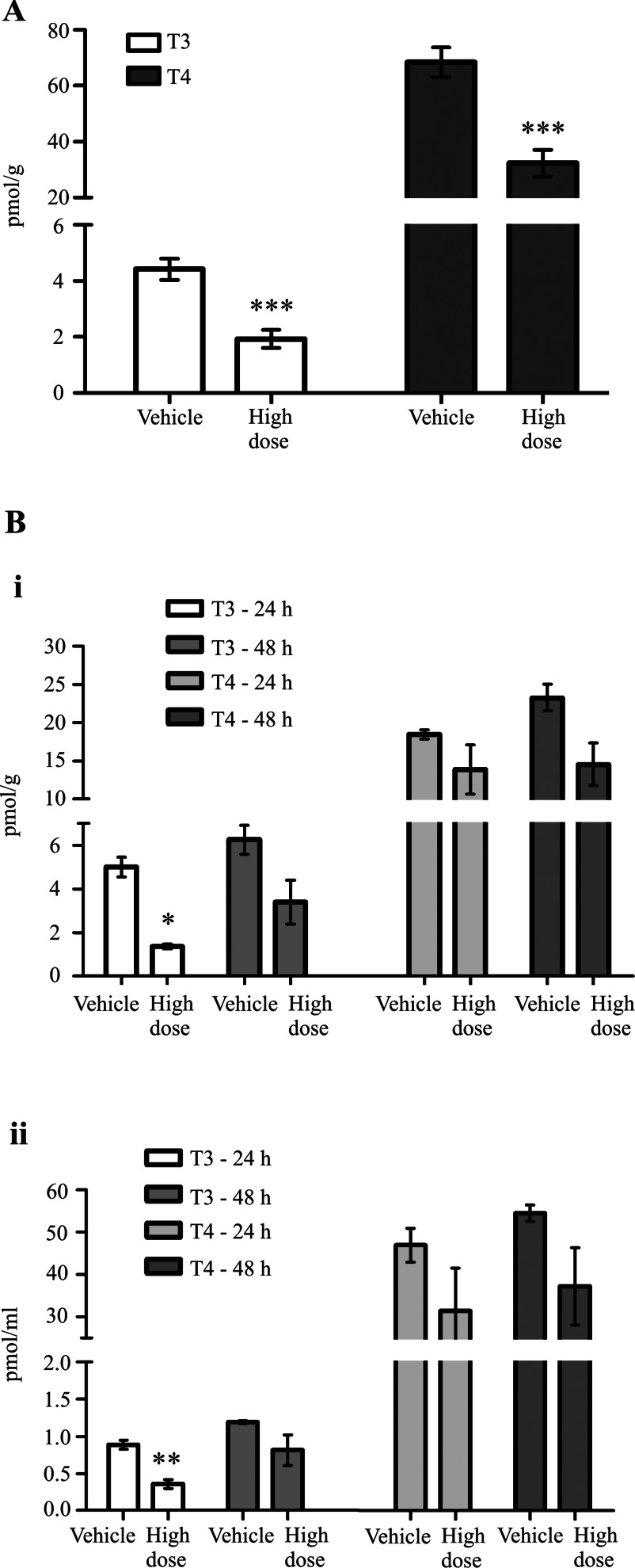
T_3_ and T_4_ levels within the liver and plasma are reduced following drug-induced liver damage Liver samples were taken for measurement using highly sensitive and specific RIAs [47,48] following high-dose treatment with (**A**) PredTox compound FP007SE and (**B**, i) thioacetamide. (**B**, ii) Plasma samples were taken for measurement as above following treatment with thioacetamide. Results are means±S.E.M. (*n*≥3). *P≤0.05, **P≤0.01, ***P≤0.001 (Student's *t* test).

We measured levels of T_3_ and T_4_ within the liver following treatment with FP007SE or thioacetamide. We saw that, following treatment with FP007SE for 15 days, there was a reduction in levels of both hormones: by 57% (T_3_) and 53% (T_4_) (Figure 7A). Similarly, thioacetamide caused a reduction in T_3_ and T_4_ levels by 73% and 25% respectively 24 h after dosing (Figure 7B, i). The levels of T_4_ were also reduced in two of the other PredTox studies (Supplementary Figure S3 at http://www.biochemj.org/bj/453/bj4530071add.htm). We also measured the levels of T_3_ and T_4_ in the plasma of the thioacetamide-treated rats. T_3_ levels were reduced by up to 60% 24 h after dosing. Although statistical significance was not achieved, T_4_ levels also showed a trend for a reduction following treatment (Figure 7B, ii). T_4_ has a long half-life in the plasma and therefore the concentration of this enzyme within the systemic circulation usually remains relatively stable [20]. Sample availability from the PredTox studies was limited, and did not allow measurement of T_3_ and T_4_ levels in the plasma.

## DISCUSSION

In the present study, we used global genomic methods to investigate changes in the rate of translation of thousands of individual mRNAs concurrently in two models of drug-induced hepatotoxicity. A change in the rate of mRNA translation can bring about a rapid efficient change in protein levels, without the need to generate more transcript [3]. We identified *Dio3* as an mRNA showing a reduction in the rate of translational activity following hepatotoxicity. The present study provides evidence that the translational repression of *Dio3* results in less D3 protein, independent of any change in mRNA level, and suggests that the reduced translation is a regulatory mechanism, enabling the liver to combat cellular stress. The known function of *Dio3* in modulating thyroxine levels supports further the hypothesis that post-transcriptional regulation may be a rapid compensatory response to drug-induced damage.

In the rat, D3 activity is reduced in cases of hypothyroidism [28,49], although the extent of the change varies between tissues [20]. The observed reductions in T_3_ and T_4_, along with the transcriptional down-regulation of *Dio1* mRNA (Supplementary Figure S4 at http://www.biochemj.org/bj/453/bj4530071add.htm), which is known to be down-regulated in cases of hypothyroidism, indicated that the livers of the treated rats were in a hypothyroid state [17,50].

Previously, changes in D3 activity have been found to correlate strongly with changes in expression of *Dio3* mRNA [49]; however, we found no evidence for the change in protein production being regulated at a transcriptional level. Moreover, one of the PredTox compounds (FP007SE) showed a transcriptional increase in *Dio3* mRNA within the liver following high-dose treatment (Figure 6A). This was in agreement with previous studies on *Dio3*, where an increase in expression was investigated [29–31]. To our knowledge, all previous work on *Dio3* in the liver has focused on cases of enhanced cellular proliferation, either during growth and development in the fetus [26] or in the adult at times of cellular stress, such as during critical illness [29]. Previous studies have assumed that the adult liver contains low to negligible levels of *Dio3* mRNA [23,50] and that, to measure transcriptional changes, cellular proliferation must first be induced. The PredTox consortium reported that, following 15 days of treatment with the FP007SE compound, there were signs of liver recovery and regeneration [51]. Furthermore, 48 h after dosing with thioacetamide, there were clinical and histological signs of liver repair, which concurred with an increase in *Dio3* mRNA levels. Therefore the results of the present study support what is widely reported in the literature whereby, in cases of cellular proliferation, *Dio3* mRNA shows an increase in expression. However, despite this increase we saw an almost total abolition of conversion of the mRNA into protein. We therefore hypothesized that the changes in protein levels were mediated predominantly by altered *Dio3* translation.

It is well established that another of the deiodinase genes, *Dio2*, is regulated at levels distinct from transcription and that these alter the levels of D2 expression independently of changes in mRNA level [37,52]. The change in activity of D2 is often greater than the change in *Dio2* mRNA, for example in brown adipose tissue following cold exposure [53] and the brain following experimentally induced hypo- and hyper-thyroidism [54]. This is essential for homoeostasis of the THs. Certainly, in cases of endoplasmic reticulum stress, D2 activity is reduced independently of transcriptional changes and this leads to a rapid, but significant, decrease in the levels of T_3_ [37].

Taken in combination, the post-transcriptional control of D2 is therefore of critical importance in maintaining TH levels in mammals. However, within the rat and human liver, D2 is not expressed [20], and, as a result, an alternative method of regulation is necessary. We hypothesize that, following hepatotoxicity, D3 is recruited for TH homoeostasis in the liver. This selenoenzyme functions primarily to inactivate T_3_ and T_4_ by conversion into their inactive metabolites, rT_3_ and T_2_ respectively [20]. As an inactivating enzyme, D3 acts primarily to protect tissues from an excess of TH. If this mechanism is disrupted in some way, severe hypothyroidism [19] or hyperthyroidism can occur.

We propose a mechanism whereby the hepatotoxins used in the present study have led to a need for increased energy for cellular proliferation, as part of the inflammatory response within the liver following cell damage. This is achieved through a rapid decrease in *Dio3* translation and consequent reduction in the level of D3 protein. Although we cannot rule out a role for increased protein degradation, the reduced ribosomal association of *Dio3* mRNA shown experimentally in the present study in both model systems provides a strong case for translational repression being an important mechanism leading to the reduction in D3 protein. In cases where *Dio3* transcription is significantly up-regulated, as seen in Figure 6, in addition to a reduction in mRNA translation, protein degradation could also contribute to the reduced D3 protein levels.

**Figure 8:**
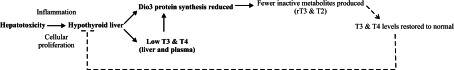
Proposed mechanism of action following drug-induced hepatotoxicity Steps in bold type are those experimentally demonstrated in the course of the present study. Non-bold type is used for the hypothesized mechanism of action and closure of the feedback loop. During hepatotoxicity, the liver is in a hypothyroid state and both the plasma and liver have reduced levels of T_3_ and T_4_. Consequently, the expression of D3 protein is reduced so that fewer inactive metabolites of T_3_ and T_4_ are produced and levels of both can return to normal, both locally (liver) and via plasma signals systemically.

The benefit of targeting *Dio3* post-transcriptionally is that the required response is efficient and rapid. A reduction in protein levels means less of the inactive metabolites rT_3_ and T_2_ are produced and alterations in T_3_ and T_4_ levels can be restored to normal (Figure 8). This is vital for maintaining the hypothalamic–pituitary–thyroid axis and means that all control is carried out at the local level, in concordance with the current literature [55]. As the liver regenerates and clinical signs of damage are reversed, protein levels are reduced further. This is probably for the required homoeostasis as the liver expends sufficient energy into tissue repair.

Although the levels of T_3_ and T_4_ within tissues are largely independent of those seen in the plasma [56], and the expression of the deiodinases is generally under localized regulation, the activity rate of the three deiodinases does directly affect the levels of circulating T_3_ and T_4_. This enables the feedback loop between the systemic organs and the thyroid gland to be maintained and has been experimentally validated in the case of D2, as described previously [37]. In the present study, we have demonstrated that, following treatment with thioacetamide, a known inducer of hypothyroidism, the levels of T_3_ and T_4_ were reduced in the plasma. The pattern of this change matched the changes in THs seen in the livers of treated animals (Figure 7B), although, as predicted by the literature [20], the change in T_4_ was smaller. D3 is located within the plasma membrane, although the bulk of the molecule is extracellular [22]. This gives it ready access to, and control over, the levels of circulating THs. We propose that, because the liver is in rapid equilibrium with the plasma, it detects a change in THs and acts rapidly to prevent further production of inactive metabolites (Figure 8). Through negative feedback, this mechanism enables levels of T_3_ and T_4_ in the liver to return to normal and ultimately restores homoeostasis within the liver.

Despite it being over 15 years since *Dio3* was first cloned [57], the regulation of this gene is complex and not yet fully understood. The gene is imprinted and preferentially expressed from the paternal allele [58]. In the mouse and humans, a non-coding gene transcribed in the antisense orientation, *Dio3os*, has been identified [59]; and very recent work indicates that a homologue of this gene may also be present in the rat [60]. *Dio3os* is proposed to belong to the family of lncRNAs (long non-coding RNAs), which, although commonly found in the mammalian genome, are poorly understood [61]. It has long been known that the stability of TRs can be moderated by naturally occurring antisense RNAs [62] and now that lncRNAs have been shown to directly regulate ribosomal association of their coding equivalent mRNAs [63], there is the distinct possibility that *Dio3* is post-transcriptionally regulated in this manner. In addition to this in the better characterized mouse and human orthologues, *Dio3os* and *Dio3* are partially overlapping and demonstrate inverse correlation of expression [59].

We conclude that the reduction in D3 expression levels during hepatotoxicity can be correlated with a change in *Dio3* mRNA translation rates. We hypothesize that this is an example of post-transcriptional control, which may be regulated by an lncRNA, *Dio3os*. We demonstrate that the magnitude of the decrease in protein is directly proportional to the extent of liver damage. This is probably due to the energy demand required for active repair processes to be initiated. TH homoeostasis has already been shown to play a role in a range of pathophysiological conditions and the results of the present study suggest that the pathway is also perturbed by drug-induced hepatotoxicity. We are able to provide evidence that this forms part of a rapid response by the liver and speculate that this occurs so that the initiation of a localized event remains consigned to the liver and does not affect the whole of the hypothalamic–pituitary–thyroid axis.

## Online data

Supplementary data
