# Supplementary material for: Decreased translation of Dio3 mRNA is associated with drug-induced hepatotoxicity
Source: Biochem J. 2013 Jun 13;453(Pt 1):71–82. doi: 10.1042/BJ20130049 (PMC3682751; doi:10.1042/BJ20130049)
Supplement: Supplementary data [file bj4530071add.pdf]

Biochem. J. (2013) **453**, 71–82 (Printed in Great Britain) doi:10.1042/BJ20130049

SUPPLEMENTARY ONLINE DATA

Decreased translation of *Dio3* mRNA is associated with drug-induced hepatotoxicity

Kate M. DUDEK*[1], Laura SUTER†, Veerle M. DARRAS‡, Emma L. MARCZYLO§ and Timothy W. GANT§[1]

*Systems Toxicology Group, Medical Research Council Toxicology Unit, University of Leicester, Lancaster Road, Leicester LE1 9HN, U.K., †Institut for Chemistry and Bioanalytics, School of Life Sciences, University of Applied Sciences and Art Northwestern Switzerland (FHNW), Mattenstrasse 22, Basel 4132, Muttenz, Switzerland, ‡Laboratory of Comparative Endocrinology, Department of Biology, Section Animal Physiology and Neurobiology, KULeuven, Naamsestraat 61, PB 2464, Leuven, B-3000, Belgium, and §Centre for Radiation, Chemical and Environmental Hazards, Public Health England, Harwell Campus, Didcot, Oxfordshire OX11 0RQ, U.K.

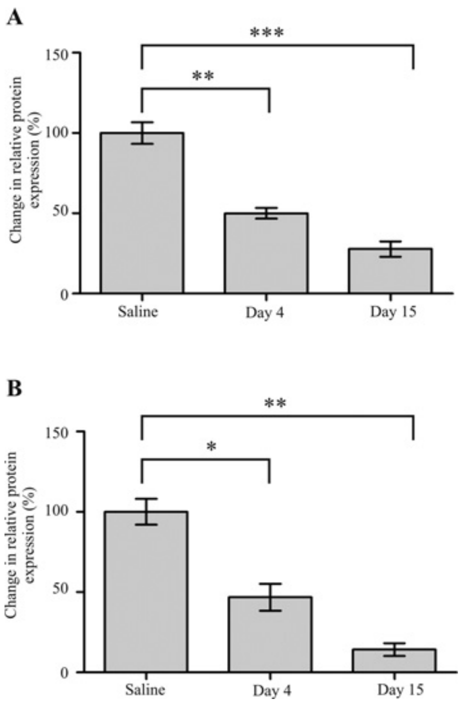

Figure S1 D3 protein is down-regulated in both a time- and dose-dependent manner

Western blot analysis was performed on lysates from the livers of rats treated with various hepatotoxic agents. D3 protein levels were normalized to those of GAPDH. Representative gel images are shown for PredTox compounds FP004BA (**A**) and FP014SC (**B**). Mean relative levels of D3 are indicated beneath each gel image. Results are means ± S.E.M. ($n = 3$). *$P \leqslant 0.05$, **$P \leqslant 0.01$, ***$P \leqslant 0.001$ (ANOVA with Tukey's/Dunnett's post-hoc test).

[1] Correspondence may be addressed to either of these authors (email kmp15@leicester.ac.uk or tim.gant@phe.gov.uk).
The full dataset for translational microarray analysis of liver samples treated with PredTox hepatotoxic compound FP014SC has been submitted to the Gene Expression Omnibus under accession number GSE38807.

© The Authors Journal compilation © 2013 Biochemical Society

Biochemical Journal www.biochemj.org

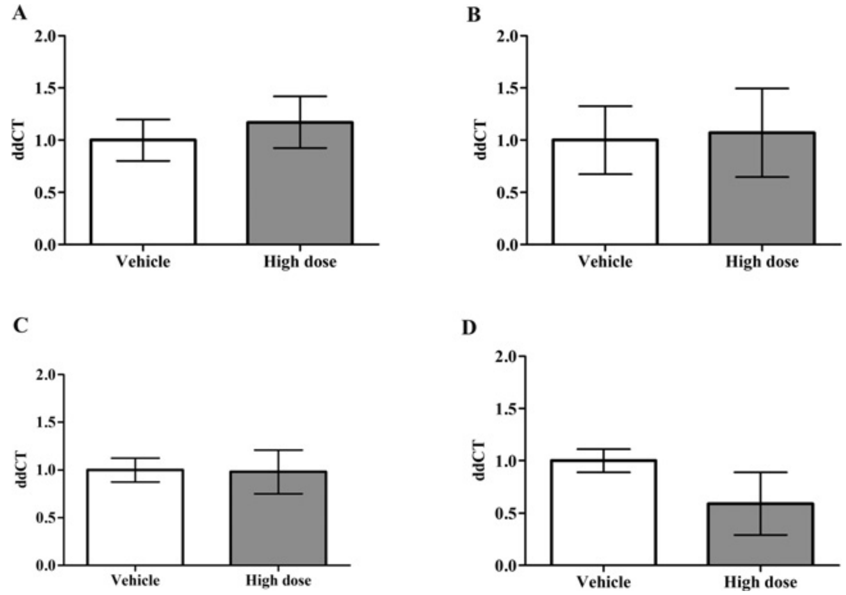

Figure S2 The reduction in D3 protein is not due to changes in mRNA transcription

qRT-PCR analysis was performed on RNA extracted from the livers of rats treated with high doses of PredTox compounds FP004BA (**A**), FP005ME (**B**), FP013NO (**C**) and FP014SC (**D**) for 15 days. Results are means ± S.E.M. ($n = 3$).

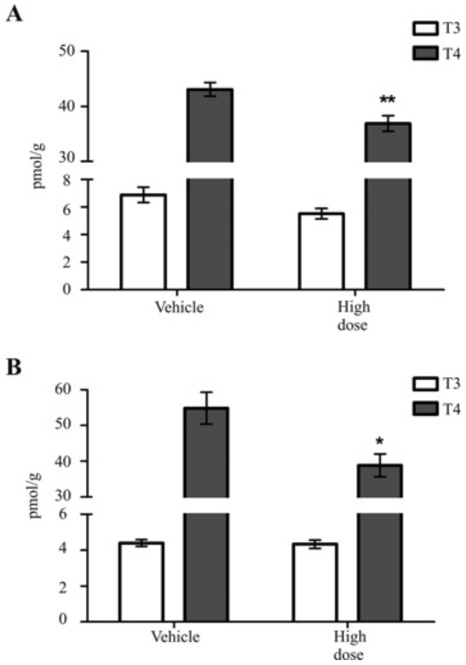

Figure S3 T_4 levels within the liver are significantly reduced following drug-induced liver damage

Liver samples were taken for measurement using highly sensitive and specific RIAs following high-dose treatment with PredTox compounds FP004BA (**A**) and FP014SC (**B**). Results represent means ± S.E.M. ($n \geqslant 3$). $^*P \leqslant 0.05$, $^{**}P \leqslant 0.01$ (Student's t test).

© The Authors Journal compilation © 2013 Biochemical Society

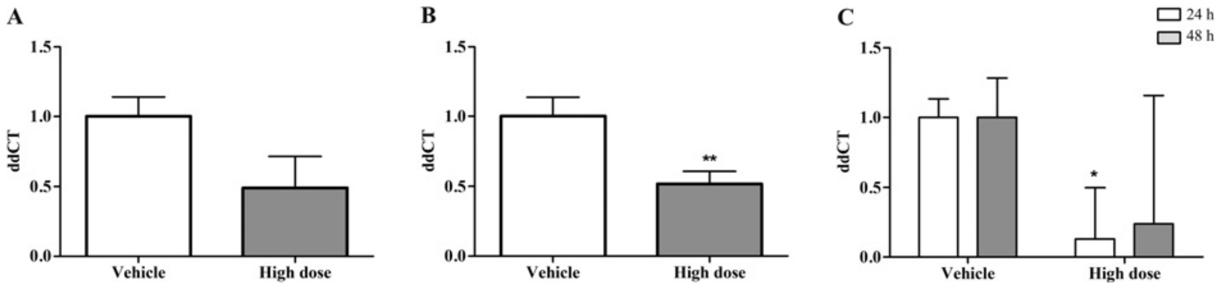

Figure S4 ***Dio1* mRNA is transcriptionally down-regulated**

qRT-PCR analysis was performed on RNA extracted from the livers of rats treated with high doses of PredTox compounds FP004BA (**A**) and FP007SE (**B**) for 15 days and (**C**) 150 mg/kg thioacetamide. Results are means $\pm$ S.E.M. ($n = 3$). $^{*}P \leqslant 0.05$, $^{**}P \leqslant 0.001$ (Student's t test).

© The Authors Journal compilation © 2013 Biochemical Society

Table S1 PredTox compounds and their properties

All available data on the five PredTox compounds selected for further analysis in the present study are provided.

Framework Programme 6 study name	Compound sponsor	Official name	Structure	Proposed high dose (mg/kg)	Proposed low dose (mg/kg)	Target organs	Chemical class	Pharmacological class
FP004BA	Bayer	(+)-(1*R*)-1-[4-(4-fluorophenyl)-2,6-di-isopropyl-5-propyl-pyridin-3-yl]ethanol	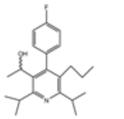	100	20	Liver, kidney	4-Phenylpyridine	Glucagon receptor antagonist
FP005ME	Merck	1-(2-Trifluoromethoxyphenyl)-2-nitroethanone	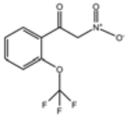	350	15	Liver	Carbonylnitromethane	Carbonylnitromethanes
FP007SE	Serono	–	–	1000	100	Kidney, liver	–	–
FP013NO	Novartis	–	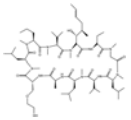	–	–	–	Cyclosporin derivative	–
FP014SC	Schering	Tetraethyl[(3-hydroxy-2-pyridyl)-amino]-methanediphosphonate	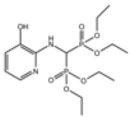	1120	280	Liver	Bisphosphonate	Inhibitor of osteoclast activity

© The Authors Journal compilation © 2013 Biochemical Society

Table S2 Primer sequences

Sequences of primers used to generate *Dio3* and *Actb* probes used in qRT-PCR and PCR experiments.

Gene	Experiment	Direction	Sequence (5′→3′)
Dio3	qRT-PCR	Forward	AGAGTGGCACCATCATGTACCA
		Reverse	CCAAGTGCGCAACTCAGACA
Dio3	PCR and sequencing	Forward	GTGGTCGGAGAAGGTGAA
		Reverse	AGAGGCGCTCAAAATAGG
Actb	qRT-PCR	Forward	CGTGAAAAGATGACCCAGATCA
		Reverse	CACAGCCTGGATGGCTACGT
Actb	PCR	Forward	ATGGTGGGTATGGGTCAGAA
		Reverse	ACATCTGCTGGAAGGTGGAC

© The Authors Journal compilation © 2013 Biochemical Society

Table S3 mRNAs changed in translational activity following treatment with PredTox compound FP014SC

Differentially translated mRNAs were identified following sucrose density fractionation and polysome profiling of liver samples treated with FP014SC. Those mRNAs that showed a statistically significant ($P \leqslant 0.05$) difference in ribosomal occupancy in at least one subpool of fractions when compared with vehicle-treated samples are listed. Significant values are denoted by bold type. *Dio3* is highlighted. Fold changes are colour-coded as indicated in the key.

Fold change
3.4
1.0
0.2

Name	Accession	Monosomes	Polysomes			Translational	Average mRNA
			Light	Medium	Heavy	change	transcript change
Aldob	NM_144903	-1.379	0.023	**0.284**	**1.759**	↑	0.17
Rbp4	NM_11255	-0.639	-0.084	0.141	**1.418**	↑	0.21
Cat	NM_9804	-0.952	**-0.572**	-0.091	**0.918**	↑	-0.17
Crp	NM_7768	**-1.022**	0.012	0.283	0.478	↑	-0.06
Nin	NM_8697	-0.842	0.070	0.033	**0.697**	↑	-0.01
1700072I22Rik		**-0.522**	0.084	0.056	**0.928**	↑	0.14
Fts	BC008130_exon	**-0.515**	0.080	**0.166**	0.839	↑	0.14
Spp2	NM_29269	-0.511	-0.186	0.097	**0.652**	↑	0.01
5930437A14Rik	NM_172542	**-0.791**	-0.099	0.032	0.395	↑	-0.12
Mug2	NM_8646	**-1.032**	-0.410	-0.204	0.049	↑	-0.40
Mug1	NM_8645	**-0.914**	-0.424	0.063	0.057	↑	-0.30
C330046E03		**-0.577**	0.101	-0.014	**0.594**	↑	0.03
Samhd1	BC067198_exon	-0.017	0.252	0.342	**1.085**	↑	0.42
Pgrmc1	NM_16783	**-0.657**	0.155	-0.111	0.543	↑	-0.02
A230072I16Rik	NM_175004	**-0.584**	**0.275**	0.046	**0.591**	↑	0.08
Kif16a		-0.179	0.138	**0.258**	**0.847**	↑	0.27
Ubb	NM_11664	0.514	0.558	**0.812**	**1.473**	↑	0.84
Pecr	NM_23523	**-0.596**	**-0.510**	-0.001	**0.265**	↑	-0.21
Mat1a	NM_133653	-0.421	**-0.449**	-0.029	0.456	↑	-0.11
C530001K22Rik		**-0.671**	-0.266	-0.153	0.306	↑	-0.20
Syt5	NM_16908	-0.081	0.134	**0.397**	**0.842**	↑	0.32
Pou4f1	NM_11143	0.254	0.084	**0.396**	**1.147**	↑	0.47
Prop1	NM_8936	**-0.356**	0.072	0.174	**0.581**	↑	0.12
Pramel4	NM_178248	0.208	0.115	0.378	**1.089**	↑	0.45
Hspa8	NM_31165	0.585	1.031	1.437	**1.391**	↑	1.11
Ass1	NM_7494	-0.301	-0.273	0.230	**0.453**	↑	0.03
Rad9	NM_11237	**-0.296**	0.119	0.213	**0.593**	↑	0.16
3110004H13Rik	NM_26068	-0.117	0.018	0.263	**0.718**	↑	0.22
4933433G15Rik		**-0.195**	0.048	0.268	**0.644**	↑	0.19
2210404O07Rik	XM_125703	0.000	0.306	0.431	**0.864**	↑	0.40
Kif21b	NM_19962	-0.471	0.107	-0.016	**0.461**	↑	0.02
4933407H18Rik	XM_132005	-0.211	-0.023	0.092	**0.635**	↑	0.12
Lu	NM_20486	**-0.556**	-0.231	-0.106	0.278	↑	-0.15
Bat1a	NM_19693	-0.337	0.105	0.026	**0.553**	↑	0.09
Imap38	NM_8376	-0.271	0.055	**0.201**	0.537	↑	0.13
9130221F21	XM_485788	0.817	0.960	0.625	**1.778**	↑	1.05
BC052055	NM_182636	-0.375	-0.040	-0.082	**0.480**	↑	0.00
Foxb1	NM_22378	-0.022	0.075	0.203	**0.773**	↑	0.26
AU016693	NM_178686	-0.299	0.023	**0.228**	0.461	↑	0.10
Tpcn1	NM_145853	-0.190	-0.021	0.125	**0.587**	↑	0.13
Sult1a1	NM_133670	-0.644	**-0.700**	-0.502	0.114	↑	-0.43
Rgs16	NM_11267	-0.350	**-0.257**	-0.025	**0.379**	↑	-0.06
Tenc1	BC042190_exon	-0.410	**0.162**	0.125	0.405	↑	0.07
unknown	AK039504_exon	-0.144	0.364	0.131	**0.732**	↑	0.27
Clecsf13	NM_16751	-0.275	-0.038	0.118	**0.470**	↑	0.07
Dhrs4	NM_30686	0.154	0.248	**0.520**	0.818	↑	0.44
Rac1	NM_9007	-0.356	0.092	0.022	**0.412**	↑	0.04
4932408F19Rik	NM_207225	-0.419	-0.111	0.004	**0.287**	↑	-0.06
Map4k6-pending	BC011346_exon	-0.182	0.248	0.161	**0.582**	↑	0.20
unknown	AF108718_exon	**-0.300**	-0.019	0.148	0.371	↑	0.05
D3Ertd300e	NM_19995	**-0.086**	0.091	0.195	0.608	↑	0.20
Camk2b	NM_7595	**-0.294**	0.119	**0.214**	**0.401**	↑	0.11
AI505034	NM_207206	-0.189	0.199	0.122	**0.559**	↑	0.17
Card10	NM_130859	-0.053	0.083	0.254	**0.611**	↑	0.22
Peli2	NM_33602	-0.211	0.090	-0.024	**0.539**	↑	0.10
Atp5g3	NM_175015	0.618	0.859	0.957	**1.292**	↑	0.93
Centg3	NM_139153	-0.105	0.039	0.221	**0.539**	↑	0.17
Cd37	NM_7645	**-0.290**	-0.029	-0.018	0.411	↑	0.02
Kcnc3	NM_8422	-0.157	0.128	**0.181**	**0.526**	↑	0.17
Slc29a4	NM_146257	-0.169	0.038	0.180	**0.468**	↑	0.13
Flii	NM_22009	-0.338	-0.037	0.032	**0.313**	↑	-0.01
Pak4	NM_27470	-0.255	0.066	**0.262**	0.347	↑	0.11
unknown	AK085517_exon	**-0.419**	-0.159	-0.200	0.278	↑	-0.12
1700039I01Rik		**-0.354**	-0.131	**-0.211**	0.333	↑	-0.09
Pip5k1c	NM_8844	-0.123	-0.011	0.162	**0.463**	↑	0.12
0910001L09Rik	XM_132434	**-0.236**	0.035	0.030	0.407	↑	0.06
C330006A16Rik		-0.177	0.179	**0.200**	0.457	↑	0.16
D11Ertd18e	NM_26740	-0.035	0.201	**0.409**	0.534	↑	0.28
A930003A15Rik		-0.223	0.054	0.113	**0.393**	↑	0.08
Chrna2	NM_144803	**-0.447**	-0.095	-0.071	0.178	↑	-0.11
Zfp162	NM_11750	0.338	0.354	**0.887**	0.790	↑	0.59
Ptprn	NM_8985	-0.287	-0.037	-0.056	**0.337**	↑	-0.01
Dyrk1b	NM_10092	-0.181	0.081	0.097	**0.422**	↑	0.10
1110059M19Rik	XM_135842	0.381	0.517	**0.477**	**0.996**	↑	0.59
Sif1		0.217	0.010	0.188	**0.757**	↑	0.29
Lgi4	NM_144556	-0.060	0.085	0.203	**0.497**	↑	0.18
Gm111	NM_207234	-0.148	**0.105**	0.076	0.455	↑	0.12
Rhbdl6	NM_172572	-0.108	0.120	**0.113**	0.484	↑	0.15
Apoc1	NM_7469	-0.685	-0.794	**-0.718**	-0.127	↑	-0.58
Aire	NM_9646	**-0.154**	**0.213**	0.161	0.445	↑	0.17
Enah	NM_10135	0.031	0.238	0.204	**0.623**	↑	0.27
unknown	AK088050_exon	**-0.214**	0.050	0.131	0.340	↑	0.08
3110007P09Rik	NM_144906	-0.326	-0.018	0.107	**0.198**	↑	-0.01
Nudt10		0.208	0.326	**0.688**	**0.645**	↑	0.47
Sts	NM_9293	-0.175	0.108	0.134	**0.374**	↑	0.11
Slc2a8	NM_19488	-0.069	0.054	0.112	**0.469**	↑	0.14
Foxk1	NM_199068	-0.184	0.090	0.078	**0.375**	↑	0.09
Pcsk2	NM_8792	-0.028	0.092	0.257	**0.472**	↑	0.20
Kif1c	NM_153103	-0.198	0.017	0.040	**0.346**	↑	0.05
Nkx6-2	NM_183248	-0.067	**0.088**	**0.173**	0.456	↑	0.16
4930471G24Rik		-0.027	0.145	**0.233**	0.493	↑	0.21
Vdp	NM_19490	**-0.304**	**-0.070**	0.009	0.217	↑	-0.04
Brd4	NM_20508	**-0.110**	0.185	0.083	0.467	↑	0.16
Mrpl24	NM_26591	0.195	0.378	**0.448**	0.715	↑	0.43
MGC68323	NM_199472	-0.008	0.224	**0.736**	0.358	↑	0.33
C030046I01Rik	NM_177994	-0.284	-0.055	-0.080	**0.259**	↑	-0.04
Ptk9l	NM_11876	-0.193	0.020	0.119	**0.304**	↑	0.06
Ppp1r14a	NM_26731	-0.004	0.230	0.171	**0.543**	↑	0.23
Pcx	NM_8797	-0.063	0.033	0.138	**0.415**	↑	0.13
Mrpl49	NM_26246	-0.079	0.198	**0.189**	0.434	↑	0.19
BC036564	XM_194421	0.127	0.088	0.229	**0.588**	↑	0.26
Amelx	NM_9666	-0.080	0.170	0.092	**0.453**	↑	0.16
Apoe	NM_9696	-0.053	0.456	0.390	**0.475**	↑	0.32
Abcf1	NM_13854	**-0.198**	0.041	0.155	**0.268**	↑	0.07
Vtn	NM_11707	**-0.736**	-0.023	-0.305	-0.141	↑	-0.30
Chdc1	XM_484393	**-0.167**	0.036	0.006	0.343	↑	0.05
2410022L05Rik	NM_25556	-0.162	-0.059	-0.041	**0.329**	↑	0.02
Krt1-24	NM_16880	**-0.283**	-0.158	-0.156	0.212	↑	-0.10
Tub	NM_21885	0.050	0.115	0.259	**0.486**	↑	0.23
Myo1a		0.150	-0.104	**-0.184**	**-0.307**	↓	-0.11
Dusp15	NM_145744	0.830	**0.371**	0.617	0.262	↓	0.52
Rps5	NM_9095	1.148	0.805	0.637	**0.718**	↓	0.83
8030442B05Rik		0.020	-0.069	-0.180	**-0.435**	↓	-0.17
Zan	NM_11741	0.188	-0.106	-0.133	**-0.300**	↓	-0.09
MoMSVgp1	NC_1502	-0.305	-0.278	**-0.444**	-0.749	↓	-0.44
Sox1	NM_9233	0.132	0.098	-0.051	**-0.320**	↓	-0.04
4930448L02Rik		0.021	-0.106	**-0.189**	-0.457	↓	-0.18
4930557B21Rik		0.027	0.014	**-0.140**	**-0.428**	↓	-0.13
1200009I06Rik	NM_28807	-0.061	-0.406	**-0.571**	-0.514	↓	-0.39
Sphk1	NM_25367	-0.034	**0.256**	-0.194	-0.392	↓	-0.09
H2-M10.1	NM_13544	**0.100**	-0.124	-0.340	-0.345	↓	-0.18
Csda	NM_11733	**0.603**	0.175	0.277	0.044	↓	0.27
Cyp11b2	NM_9991	-0.166	-0.411	**-0.390**	-0.702	↓	-0.42
Hrg	NM_53176	**-0.465**	**-0.343**	-0.679	-0.887	↓	-0.59
Smpd2	NM_9213	0.066	-0.078	**-0.151**	**-0.446**	↓	-0.15
2610034P21Rik		-0.017	-0.056	**-0.290**	-0.481	↓	-0.21
Gfi1	NM_10278	0.191	-0.143	0.018	**-0.409**	↓	-0.09
Ptgdr	NM_8962	1.087	0.879	**0.727**	0.590	↓	0.82
4631403P03Rik	NM_153793	-0.071	-0.219	**-0.484**	**-0.531**	↓	-0.33
Acox3	NM_30721	-0.049	-0.057	**-0.188**	-0.555	↓	-0.21
Gnb2-rs1	NM_8143	**0.450**	0.094	0.088	-0.100	↓	0.13
D15Wsu169e	NM_198420	0.080	-0.116	**-0.154**	-0.460	↓	-0.16
Mup1	NM_31188	**-1.238**	**-1.816**	**-2.335**	**-1.619**	↓	-1.75
Hist3h2ba	NM_30082	**0.208**	-0.167	0.044	-0.417	↓	-0.08
Bcl6b	NM_7528	**0.351**	0.375	-0.034	-0.071	↓	0.16
AW547186	NM_177592	-0.042	-0.042	-0.082	**-0.589**	↓	-0.19
Tnnt3	NM_11620	0.049	-0.117	**-0.339**	-0.439	↓	-0.21
Atf1	NM_7497	**-0.139**	-0.201	**-0.333**	-0.660	↓	-0.33
unknown	BC060148_exon	-0.123	-0.230	**-0.203**	-0.700	↓	-0.31
4930421P22Rik		**0.224**	0.026	-0.034	-0.338	↓	-0.03
Centa1	NM_172723	0.073	-0.037	**-0.349**	-0.410	↓	-0.18
1110020A09Rik	NM_27415	-0.191	-0.278	**-0.319**	**-0.768**	↓	-0.39
Zcchc3	NM_175126	-0.243	-0.361	**-0.382**	**-0.833**	↓	-0.45
B4galt2	NM_17377	-0.132	-0.116	**-0.378**	-0.644	↓	-0.32
5133400G04Rik	NM_27733	0.028	-0.053	**-0.064**	**-0.579**	↓	-0.17
4930405A07Rik		-0.026	-0.055	**-0.245**	-0.575	↓	-0.23
Fetub	NM_21564	**-0.329**	-0.419	**-0.729**	**-0.839**	↓	-0.58
Tjp3	NM_13769	0.072	-0.225	**-0.152**	-0.567	↓	-0.22
Ttl	NM_27192	0.096	-0.172	**-0.164**	-0.537	↓	-0.19
Mup4	NM_8648	**-1.181**	-1.653	**-2.342**	**-1.597**	↓	-1.69
2010012O05Rik	NM_25563	-0.001	-0.119	-0.106	**-0.655**	↓	-0.22
2300003C06Rik	NM_28001	**0.221**	0.184	-0.053	-0.351	↓	0.00
Fgf23	NM_22657	0.206	0.111	-0.042	**-0.396**	↓	-0.03
Foxo6	NM_194060	-0.030	-0.120	**-0.120**	-0.690	↓	-0.24
2510015F01Rik	XM_354801	0.120	0.070	**-0.119**	-0.480	↓	-0.10
Cyp2c50	NM_134144	0.242	0.167	-0.126	**-0.328**	↓	-0.01
Tubb4	NM_9451	-0.451	-0.513	**-0.522**	**-1.124**	↓	-0.65
Notch4	NM_10929	0.088	0.016	-0.148	**-0.541**	↓	-0.15
5830428M24Rik	XM_488638	0.050	-0.160	**-0.247**	-0.612	↓	-0.24
Rpl21	NM_19647	1.219	1.236	0.723	**0.699**	↓	0.97
4930441N18Rik		0.036	-0.112	**-0.373**	**-0.573**	↓	-0.26
Nr2f6	NM_10150	-0.137	-0.334	**-0.410**	-0.809	↓	-0.42
r171.1	NC_2512	0.028	-0.122	**-0.260**	-0.630	↓	-0.25
Banf1	NM_11793	-0.137	-0.124	**-0.346**	-0.769	↓	-0.34
D5Ertd708e	NM_24216	**0.282**	-0.028	-0.071	-0.413	↓	-0.06
3110052M02Rik	XM_484601	-0.370	-0.173	-0.254	**-1.071**	↓	-0.47
Mycs	NM_10850	-0.018	-0.102	-0.199	**-0.723**	↓	-0.26
1110007A13Rik	NM_145955	0.024	-0.109	**-0.169**	**-0.700**	↓	-0.24
Pold1	NM_11131	**0.259**	-0.191	-0.199	-0.483	↓	-0.15
Cyp2j13	NM_145548	**-0.444**	-1.054	**-1.297**	-1.118	↓	-0.98
Park2	NM_16694	-0.216	-0.363	**-0.565**	-0.910	↓	-0.51
Zcchc3	NM_175126	-0.069	-0.219	-0.263	**-0.821**	↓	-0.34
Sumo1	NM_9460	-0.174	-0.208	-0.022	**-1.009**	↓	-0.35
Zdhhc21	NM_26647	-0.017	-0.303	**-0.487**	-0.736	↓	-0.39
A930033C23Rik		0.108	0.011	-0.084	**-0.649**	↓	-0.15
2410187C16Rik	XM_128102	**0.914**	0.131	0.195	0.075	↓	0.33
2610301G19Rik	BC021475_exon	-0.229	-0.253	**-0.357**	-1.016	↓	-0.46
3110052M02Rik	XM_484601	-0.006	-0.372	-0.151	**-0.904**	↓	-0.36
Eif3s4	NM_16876	**0.120**	-0.024	-0.189	-0.675	↓	-0.19
Gja9	NM_10290	**0.287**	-0.159	-0.128	-0.583	↓	-0.15
3110009E18Rik	NM_28439	-0.073	**-0.379**	**-0.237**	-0.987	↓	-0.42
unknown	BC065789_exon	0.006	-0.135	**-0.205**	-0.824	↓	-0.29
4930563H03Rik		-0.352	-0.680	**-0.915**	-1.175	↓	-0.78
Plvap	NM_32398	-0.072	-1.015	**-0.595**	-1.120	↓	-0.70
Uchl1	NM_11670	-0.010	-0.052	**-0.293**	**-0.874**	↓	-0.31
4933434G05Rik	XM_126681	-0.066	-0.212	-0.131	**-1.078**	↓	-0.37
Orc5l	NM_11959	**0.162**	-0.296	-0.119	-0.895	↓	-0.29
4930438O05Rik	NM_27507	0.103	-0.221	-0.130	**-0.939**	↓	-0.30
2410080H04Rik	NM_172522	0.066	-0.594	**-0.341**	-1.055	↓	-0.48
Arl2bp	NM_24191	0.155	-0.261	0.009	**-0.978**	↓	-0.27
Tcirg1	NM_16921	-0.210	-0.381	**-0.472**	-1.246	↓	-0.58
Pin1	NM_23371	0.223	-0.181	-0.109	**-0.940**	↓	-0.25
Narfl	NM_26238	**0.260**	-0.565	**-0.167**	-1.026	↓	-0.37
B230208J24Rik	NM_145356	-0.136	-0.443	**-0.407**	-1.309	↓	-0.57
Dio3	NM_172119	-0.396	-0.602	**-0.775**	-1.508	↓	-0.82
1700095D23Rik		0.128	-0.315	**-0.476**	-1.053	↓	-0.43
Fstl1	NM_8047	-0.078	**-0.517**	**-0.810**	-1.268	↓	-0.67
Serpina3c	NM_8458	0.215	-0.169	**-0.809**	**-0.927**	↓	-0.42
Arrdc2	XM_134227	-0.012	-0.292	-0.197	**-1.478**	↓	-0.49
Pacs1	NM_153129	-0.042	-0.143	-0.334	**-1.435**	↓	-0.49
Cmkor1	NM_7722	0.241	**-0.233**	0.131	-1.447	↓	-0.33

© The Authors Journal compilation © 2013 Biochemical Society

Table S4 Canonical pathways that were significantly perturbed (two or more genes) following treatment with PredTox compound FP014SC

All mRNAs that showed a change in ribosomal occupancy across the gradient following polysome profiling were uploaded for pathway analysis to Ingenuity® IPA software. Those pathways under the control of RXR are in bold (FXR, farnesoid X receptor; IL-1, interleukin 1; LPS, lipopolysaccharide; LXR, liver X receptor; PPARα, peroxisome-proliferator-activated receptor α; PXR, pregnane X receptor).

Ingenuity® canonical pathway	− log (*P* value)	Number of altered genes
Fatty acid metabolism	8.71	15
**LPS/IL-1 mediated inhibition of RXR function**	2.67	10
Production of nitric oxide and reactive oxygen species in macrophages	3.10	10
Clathrin-mediated endocytosis signalling	2.99	10
G-protein-coupled receptor signalling	0.319	9
mTOR signalling	2.47	9
Axonal guidance signalling	0.689	9
Glucocorticoid receptor signalling	1.51	9
Tryptophan metabolism	2.81	8
Protein kinase A signalling	0.834	8
**PPARα/RXRα activation**	2.11	8
Metabolism of xenobiotics by cytochrome P450	3.18	8
**LXR/RXR activation**	3.15	8
RAR activation	2.11	8
eIF2 signalling	1.94	8
Regulation of eIF4 and p70^{S6K} signalling	2.57	8
Protein ubiquitination pathway	1.16	8
PI3K signalling in B-lymphocytes	2.14	7
Role of macrophages, fibroblasts and endothelial cells in rheumatoid arthritis	0.613	7
Molecular mechanisms of cancer	0.475	7
Calcium signalling	1.58	7
Xenobiotic metabolism signalling	0.718	7
Dopamine–DARPP32 feedback in cAMP signalling	1.71	7
ERK/MAPK signalling	1.47	7
Acute-phase response signalling	1.67	7
Valine, leucine and isoleucine degradation	3.11	6
CREB signalling in neurons	1.17	6
B-cell receptor signalling	1.48	6
Breast cancer regulation by Stathmin1	0.981	6
Wnt β-catenin signalling	1.19	6
Bile acid biosynthesis	3.58	6
Arachidonic acid metabolism	1.75	6
RhoGDI signalling	0.773	5
Ephrin receptor signalling	0.794	5
Huntington's disease signalling	0.530	5
Purine metabolism	0.330	5
Linoleic acid metabolism	2.07	5
Glycolysis/gluconeogenesis	1.81	5
Fatty acid elongation in mitochondria	4.93	5
Phospholipase C signalling	0.468	5
Atherosclerosis signalling	1.33	5
IL-12 signalling and production in macrophages	1.15	5
Actin cytoskeleton signalling	0.554	5
Sertoli cell–Sertoli cell junction signalling	0.745	5
Semaphorin signalling in neurons	1.97	4
NRF2-mediated oxidative stress response	0.457	4
P2Y purigenic receptor signalling pathway	0.848	4
Tyrosine metabolism	1.46	4
Pyruvate metabolism	1.62	4
Human embryonic stem cell pluripotency	0.753	4
GNRH signalling	0.821	4
Role of NFAT in cardiac hypertrophy	0.439	4
Synaptic long-term potentiation	1.02	4
Colorectal cancer metastasis signalling	0.250	4
Glioblastoma multiforme signalling	0.623	4
PI3K/Akt signalling	0.867	4
PTEN signalling	0.979	4
Hereditary breast cancer signalling	0.886	4
Tight junction signalling	0.585	4
IL-8 signalling	0.491	4
Glycerolipid metabolism	1.05	4
**FXR/RXR activation**	1.25	4
Inositol phosphate metabolism	0.616	4
AMPK signalling	0.737	4
Mitochondrial dysfunction	0.737	4
Type 2 diabetes mellitus signalling	0.906	4

© The Authors Journal compilation © 2013 Biochemical Society

Table S4 Continued

Ingenuity® canonical pathway	− log (*P* value)	Number of altered genes
Ceramide signalling	1.39	4
Aldosterone signalling in epithelial cells	0.579	4
Amyotrophic lateral sclerosis signalling	1.08	4
Signalling by Rho family GTPases	0.266	4
Sphingosine 1-phosphate signalling	0.926	4
Germ cell–Sertoli cell junction signalling	0.573	4
p38 MAPK signalling	0.590	3
Circadian rhythm signalling	1.84	3
FGF signalling	0.787	3
Pentose and glucuronate interconversions	1.55	3
GM-CSF signalling	1.08	3
Neuropathic pain signalling in dorsal horn neurons	0.605	3
iCOS–iCOSL signalling in T-helper cells	0.590	3
PKCθ signalling in T-lymphocytes	0.526	3
Prostate cancer signalling	0.820	3
Cyclins and cell-cycle regulation	0.843	3
Aryl hydrocarbon receptor signalling	0.401	3
Small-cell lung cancer signalling	0.944	3
Thyroid cancer signalling	1.44	3
Non-small-cell lung cancer signalling	1.03	3
Retinol metabolism	1.44	3
**TR/RXR activation**	0.777	3
Galactose metabolism	1.47	3
Alanine and aspartate metabolism	1.55	3
Dendritic cell maturation	0.304	3
OX40 signalling pathway	0.766	3
Cdc42 signalling	0.293	3
Type 1 diabetes mellitus signalling	0.567	3
Insulin receptor signalling	0.437	3
Natural killer cell signalling	0.605	3
Oxidative phosphorylation	0.368	3
Role of Oct4 in mammalian embryonic stem cell pluripotency	1.42	3
Cardiac β-adrenergic signalling	0.426	3
fMLP signalling in neutrophils	0.575	3
CCR3 signalling in eosinophils	0.533	3
CXCR4 signalling	0.330	3
Paxillin signalling	0.629	3
LPS-stimulated MAPK signalling	0.917	3
Regulation of actin-based motility by Rho	0.787	3
Rac signalling	0.605	3
$p70^{S6K}$ signalling	0.476	3
NGF signalling	0.605	3
Androgen and oestrogen metabolism	0.832	3
Sulfur metabolism	2.38	3
Fructose and mannose metabolism	1.30	3
Renal cell carcinoma signalling	0.958	3
Hypoxia signalling in the cardiovascular system	1.00	3
RhoA signalling	0.258	2
Neurotrophin/Trk signalling	0.498	2
ATM signalling	0.671	2
Arginine and proline metabolism	0.466	2
Ubiquinone biosynthesis	0.490	2
Basal cell carcinoma signalling	0.490	2
Factors promoting cardiogenesis in vertebrates	0.372	2
Role of Nanog in mammalian embryonic stem cell pluripotency	0.266	2
Glioma signalling	0.329	2
Melatonin signalling	0.474	2
Urea cycle and metabolism of amino groups	1.01	2
Oestrogen-dependent breast cancer signalling	0.581	2
p53 signalling	0.339	2
Bladder cancer signalling	0.361	2
Androgen signalling	0.254	2
Cell cycle: G_1/S checkpoint regulation	0.624	2
Chronic myeloid leukaemia signalling	0.334	2
Cell cycle regulation by BTG family proteins	0.942	2
HGF signalling	0.309	2
Pancreatic adenocarcinoma signalling	0.278	2
**PXR/RXR activation**	0.498	2
IL-1 signalling	0.344	2
Glutamate receptor signalling	0.613	2

© The Authors Journal compilation © 2013 Biochemical Society

Table S4 Continued

Ingenuity® canonical pathway	− log (*P* value)	Number of altered genes
Butanoate metabolism	0.581	2
Telomerase signalling	0.305	2
Cross-talk between dendritic cells and natural killer cells	0.355	2
Cytotoxic T-lymphocyte-mediated apoptosis of target cells	0.409	2
Allograft rejection signalling	0.397	2
Caveolae-mediated endocytosis signalling	0.458	2
Virus entry via endocytic pathways	0.355	2
Neuroprotective role of THOP1 in Alzheimer's disease	0.848	2
FcεRI signalling	0.291	2
PDGF signalling	0.482	2
CD40 signalling	0.552	2
TNFR1 signalling	0.724	2
Angiopoietin signalling	0.533	2
Parkinson's disease signalling	1.45	2
Apoptosis signalling	0.361	2
Retinoic acid-mediated apoptosis signalling	0.552	2
Citrate cycle	1.08	2
Activation of IRF by cytosolic pattern recognition receptors	0.552	2
Dopamine receptor signalling	0.444	2
CDK5 signalling	0.372	2
Regulation of IL-2 expression in activated and anergic T-lymphocytes	0.423	2
CD28 signalling in T-helper cells	0.240	2
Agrin interactions at neuromuscular junctions	0.507	2
FAK signalling	0.372	2
PAK signalling	0.361	2
Renin–angiotensin signalling	0.266	2
Role of BRCA1 in DNA damage response	0.624	2
Sphingolipid metabolism	0.397	2
Keratan sulfate biosynthesis	0.635	2
Inositol metabolism	2.41	2
G-protein signalling mediated by Tubby	0.963	2
14-3-3-mediated signalling	0.226	2
Starch and sucrose metabolism	0.507	2

Received 9 January 2013/12 April 2013; accepted 16 April 2013
Published as BJ Immediate Publication 16 April 2013, doi:10.1042/BJ20130049

© The Authors Journal compilation © 2013 Biochemical Society
